# Supplementary material for: Influence of Some Spaghetti Processing Variables on Technological Attributes and the In Vitro Digestion of Starch
Source: Foods. 2022 Nov 15;11(22):3650. doi: 10.3390/foods11223650 (PMC9689111; doi:10.3390/foods11223650)
Supplement: Supplementary file 1 [file foods-11-03650-s001.zip › foods-2009154-supplementary.pdf]

Table S1. Extrusion variables evaluated and conditions used. Powder feed rate, added water, screw speed, torque, power consumption, die temperature and pasta moisture during the extrusion trial

Sample No.	Feed rate (g/min)	Water addition (mL/min)	Screw speed (rpm)	Torque (Nm)	Pressure (bar)	Die Temp. (°C)	Power (kW)	Pasta %M
1	40	15	300	4	26	60	0.12	28.53
2	40	15	250	4.3	27	58	0.1	nd
3	40	15	200	5	27	54	0.1	29.17
4	40	15	150	13	34	52	0.2	nd
6	44	15	200	5.5	32	59	0.12	nd
5	40	15	200	4.5	28	50	0.09	nd
7	36	15	200	3.1	22	60	0.06	nd
8	32	15	200	2.2	14	54	0.04	nd
13	40	15	200	4	27	61	0.1	27.39
14	40	15	200	4.5	26	70	0.09	24.41
15	48	15	200	7.5	35	75	0.15	22.92
16	48	15	200	6	25	85	0.12	21.04

Nd=not determined.
